# Systematic Evaluation of Zn^2+^, Ca^2+^, and Co^2+^ Doping for Tailoring the Thermal, Structural, Morphological and Magnetic Performance of CdBi_0.1_Fe_1.9_O_4_@SiO_2_ Nanocomposites

**DOI:** 10.3390/nano16040259

**Published:** 2026-02-16

**Authors:** Thomas Dippong, Ioan Petean, Oana Cadar

**Affiliations:** 1 Faculty of Science, Technical University of Cluj-Napoca, 76 Victoriei Street, 430122 Baia Mare, Romania; 2 Faculty of Chemistry and Chemical Engineering, Babes-Bolyai University, 11 Arany Janos Street, 400084 Cluj-Napoca, Romania; ioan.petean@ubbcluj.ro; 3 INCDO-INOE 2000, Research Institute for Analytical Instrumentation, 67 Donath Street, 400293 Cluj-Napoca, Romania; oana.cadar@icia.ro

**Keywords:** cadmium-bismuth doped ferrite, divalent ions, SiO_2_ matrix, structural-property relationship, magnetic characteristics

## Abstract

The influence of Zn^2+^, Ca^2+^ and Co^2+^ doping on the thermal, structural, morphological, and magnetic characteristics of CdBi_0.1_Fe_1.9_O_4_ nanoparticles synthetized via the sol–gel technique and calcined at 300, 600, 900 and 1200 °C was investigated. Thermal analysis revealed the initial formation of metallic glyoxylates up to 300 °C, followed by their decomposition into metal oxides and subsequent ferrite formation. X-ray diffraction revealed that the ferrites were poorly crystallized at lower temperatures, whereas at higher calcination temperatures all nanocomposites exhibited well-crystalized ferrites coexisting with the SiO_2_ matrix, except for the Co_0.1_Cd_0.9_Bi_0.1_Fe_1.9_O_4_@SiO_2_ nanocomposite, which formed a single, well-defined crystalline phase. Atomic force microscopy images revealed spherical ferrite particles encapsulated within an amorphous layer, with particle size, surface area, and coating thickness influenced by both the type of dopant ion and the calcination temperature. The structural parameters estimated by X-ray diffraction, as well as the magnetic characteristics, were strongly influenced by the dopant type and thermal treatment. These results demonstrate that the structural and magnetic characteristics of CdBi_0_._1_Fe_1_._9_O_4_ ferrites can be effectively tuned through controlled doping and calcination, providing insights for the design of tailored functional applications.

## 1. Introduction

Nanostructured ferrites exhibit advantageous magnetic characteristics, such as reduced coercivity (*H_c_*), high saturation magnetization (*M_s_*), elevated magnetic permeability, large surface area, high electrical resistivity, and low dielectric and eddy current losses. In addition, their mechanical rigidity, chemical and thermal stability, non-toxicity, physical flexibility, corrosion resistance, and cost effectiveness further enhance their technological significance [1,2,3,4,5,6,7,8,9]. Nevertheless, the structural and magnetic characteristics of ferrites are strongly dependent on their chemical composition, precursor selection, synthesis strategy, thermal processing conditions, and the incorporation of dopants, impurities, or secondary phases [6,7,8,9,10,11,12]. Owing to their multifunctional characteristics, ferrites find applications across several technological sectors, including (i) biomedical applications such as anticancer and antibacterial therapies, bioimaging, biosensors), (ii) energy and environmental applications, including energy storage, microwave absorption, magnetic refrigeration, corrosion protection, and water decontamination, (iii) electronic and magnetic technologies, such as spintronics, humidity sensors, ferrofluids, high-capacity information storage media, and (iv) industrial applications, including ceramic pigments, ferroceramics, and magnetic inks [13,14,15,16,17,18].

CdFe_2_O_4_ crystallizes in a normal spinel structure, in which Cd^2+^ ions preferentially occupy the tetrahedral (A) sites and Fe^3+^ ions on the octahedral (B) sites within a face-centered cubic lattice [1,2,3,4]. Moreover, CdFe_2_O_4_ is a typical example of geometrically frustrated antiferromagnet, exhibiting unique magnetic behavior that arise from superexchange interactions among its magnetic ions [1,2,3,4,5]. Although CdFe_2_O_4_ is non-magnetic, the relatively large radius of the Cd^2+^ ion induces a lattice distortion in the spinel structure, which can lead to spin canting and perturbation of the magnetic moment [1,3,5,8]. Compared to other magnetic materials, CdFe_2_O_4_ exhibits high electrical resistivity, a characteristic that is particularly advantageous for the design of soft ferrites, as it minimizes eddy-current losses and provides a clear advantage over magnetic materials such as metal alloys and metal oxides [1,2,3,4,5,6,7]. Owing to the synergy of its chemical stability, structural, mechanical integrity, and multifunctional electrical, magnetic, and optical characteristics, CdFe_2_O_4_ is suitable for a wide range of applications including transformer core materials, microwave components, and biomedical technologies [1,2,3,4,5,6].

The incorporation of non-magnetic Cd^2+^ ions into the spinel ferrite lattice is known to modify the cation distribution and enhance magnetic performance, notably by increasing the net magnetic moment and *M_s_*, enabling applications in loading coils, magnetic recording heads, antenna cores and microwave devices [3,5,6]. Furthermore, co-doping with divalent ions Zn^2+^ or Co^2+^ can further improve magnetic characteristics, electrical conductivity, permeability, density, photocatalytic activity, and biological activity [7,8,9]. Additionally, substituting Bi^3+^ or Fe^3+^ ions with other transition metal ions can induce lattice distortions, modify the cycloidal spin structure and enhance ferrimagnetism [19,20,21,22].

Numerous synthetic strategies have been developed for ferrite preparation, including wet chemical techniques (co-precipitation, hydrothermal/solvothermal methods), sonochemical, sol-gel, solid-state methods (solid-state reaction, ball milling/mechanochemical processing), mechanical treatments (milling, grinding), thermal and combustion-based methods (decomposition, flash combustion, auto-combustion), as well as vapor phase and aerosol-assisted techniques (vapor phase deposition and surfactant-mediated aerosol processing) [23,24,25,26,27,28,29]. Each method provides distinct advantages in tuning particle size, morphology, and functional characteristics, with wet-chemical routes favoring nanosized particles and solid-state routes typically used for bulk ferrite systems [23,27,28]. Among these approaches, the sol-gel method provides a versatile and cost-effective strategy for producing ferrites with well-controlled composition, structural uniformity, and tailored functional characteristics [23,27,28,29,30,31]. Nanoparticle agglomeration, which can hinder the formation of single-phase ferrites, can be mitigated by applying homogenous ultrathin coatings or dispersing particles within non-magnetic matrices. In this context, the sol-gel method using tetraethylorthosilicate (TEOS) enables the formation of robust inorganic networks with moderate reactivity, facilitating the incorporation of a wide range of organic and inorganic species. Among potential coating materials, mesoporous SiO_2_ is particularly attractive due to its hydrophilicity, chemical stability, non-toxicity and biocompatibility, and its ability to control particle growth and size distribution [23,27,28]. Embedding ferrite nanoparticles within a SiO_2_ matrix suppresses agglomeration, promotes controlled crystallite development, and can enhance magnetic performance. Moreover, the bio-inert SiO_2_ matrix improves the biocompatibility of nanoferrites and mitigates inflammatory risks in biological environments [23,27,28].

The magnetic behavior of nanoferrites is primarily determined by the combined effects of cation distribution at the A and B sites and surface-related spin disorder, both of which are influenced by particle size, synthesis method, and post-synthesis thermal processing conditions [32,33,34]. Additionally, the magnetic characteristics of CoFe_2_O_4_, ZnFe_2_O_4_ and CdFe_2_O_4_ are influenced by size, shape, purity, and magnetic stability of the constituent particles [27]. Multiferroic systems have attracted significant interest due to the coexistence of switchable ferroelectric, ferrimagnetic, and ferroelastic order parameters within a single phase [19,35]. However, when ferrimagnetic ferrite particles decrease below a critical size, thermal fluctuations can disrupt single-domain magnetic ordering, leading to a substantially reduction in *M_s_* and coercivity (*H_c_*) [27].

This study investigates the influence of Zn^2+^ (Zn_0.1_Cd_0.9_Bi_0.1_Fe_1.9_O_4_), Ca^2+^ (Ca_0.1_Cod_0.9_Bi_0.1_Fe_1.9_O_4_), and Co^2+^ (Co_0.1_Cd_0.9_Bi_0.1_Fe_1.9_O_4_) doping on the structural, morphological, and magnetic characteristics of CdBi_0.1_Fe_1.9_O_4_ nanoparticles embedded within a SiO_2_ matrix, synthetized via the sol-gel route and calcined at various temperatures. The thermal evolution and decomposition of glyoxylate precursors were analyzed using thermal analysis and Fourier transform infrared (FT-IR) spectroscopy. The crystalline phase development and crystallinity were assessed using X-ray diffraction (XRD) which was used to study, while particle shape, morphology, size and agglomeration were investigated using atomic force microscopy (AFM). The magnetic characteristics, including the variation of *M_s_* vs. *H_c_* were evaluated for both undoped and Zn-, Ca- and Co-doped CdBi_0.1_Fe_1.9_O_4_ samples.

The novelty of the present study arises from (i) a comprehensive assessment of the combined effect of the SiO_2_ matrix and divalent dopant ions on the formation of metal-glyoxylate precursors and their subsequent thermal transformation into ferrite phases embedded in the SiO_2_ matrix; (ii) a systematic comparative investigation of Zn^2+^, Ca^2+^, and Co^2+^ doping in CdBi_0.1_Fe_1.9_O_4_@SiO_2_ nanocomposites (NCs) to elucidate their influence on morphological, structural, and magnetic characteristics, identifying pathways to enhance functional performance and broaden potential applications; (iii) the development of new trimetallic ferrites embedded in a SiO_2_ matrix; (iv) achieving single-phase crystalline ferrites at relatively low calcination temperatures (300 °C) for both undoped and doped systems, demonstrating the efficiency of the proposed synthesis; and (v) showing that dopant incorporation, particularly Co^2+^ ion, suppresses the formation of secondary phases at high calcination temperatures (1200 °C), highlighting the stabilizing role of selective doping.

## 2. Materials and Methods

### 2.1. Reagents

Zinc nitrate hexahydrate (Zn(NO_3_)_2_∙6H_2_O, 98%), calcium nitrate tetrahydrate (Ca(NO_3_)_2_∙4H_2_O, 99%), cobalt nitrate hexahydrate (Co(NO_3_)_2_∙6H_2_O, 98%), and cadmium nitrate tetrahydrate (Cd(NO_3_)_2_∙4H_2_O, 99%), were purchased from Carlo Erba (Milan, Italy). Ferric nitrate nonahydrate (Fe(NO_3_)_3_∙9H_2_O, 98%), bismuth nitrate pentahydrate (Bi(NO_3_)_2_·5H_2_O (98%)), ethylene glycol (EG, 99%), TEOS (99%) and ethanol (96%) were obtained from Merck, Darmstadt, Germany.

### 2.2. Synthesis

CdBi_0.1_Fe_1.9_O_4_ and M_0.1_Cd_0.9_Bi_0.1_Fe_1.9_O_4_ (M = Zn, Ca, Co) embedded within a SiO_2_ matrix were prepared using a sol-gel technique. The resulting NCs consisted of 45 wt.% ferrite and 55 wt.% SiO_2_. Appropriate molar ratios of metal nitrates were selected to achieve M/Cd/Bi/Fe of 0/10/1/19 for the undoped composition and 1/9/1//19 for the Zn-, Ca-, and Co-doped samples. The precursor sols were prepared by dissolving the corresponding metal nitrates in a mixed solvent system containing EG, TEOS, and ethanol using a NO_3_^−^/EG/TEOS molar ratio of 1/1/1. The solutions were magnetically stirred for 30 min to ensure homogeneity and subsequently aged under ambient conditions until complete gelation, which occurred after approximately 30 days. The resulting gels entrapped uniformly distributed metal ions within the organic–inorganic network. To enhance the phase purity and promote the crystallite growth, the dried gels were finely ground and subjected to a thermal pretreatment at 40 °C for 4 h. Calcination was then carried out subsequentially at 300, 600, 900 and 1200 °C for 4 h at each temperature using a Nabertherm LT9 muffle furnace (Lilienthal, Germany).

### 2.3. Characterization

The thermal evolution of the samples was investigated by thermogravimetric (TG) and differential thermal analysis (DTA) in air, using a heating rate of at 10 °C·min^−1^ up to 1200 °C; measurements were performed SDT Q600 analyzer (TA Instruments, New Castle, DE, USA), employing alumina as the reference material. FT-IR spectra were recorded on KBr pellets containing 1 wt.% of the sample using a Prestige-21 (Shimadzu, Tokyo, Japan). Phase identification was performed by XRD using a D8 Advance diffractometer (Bruker, Karlsruhe, Germany) with CuKα radiation (λ = 1.5418 Å) operated at 40 kV and 35 mA. Atomic force microscopy (AFM) measurements were performed using a high-resolution scanning probe microscope JSPM 4210 produced by JEOL Company (Tokyo, Japan) operating in tapping mode with a NSC 15 Hard cantilever (resonant frequency of 325 kHz and a force constant of 40 N/m). The acquired topographic images were analyzed with WinSPM software (version 2.0, JEOL, Tokyo, Japan) to determine surface height, roughness, and particle diameter. The magnetic characterization was carried out using a 7400 vibrating-sample magnetometer (VSM, Lake Shore, Carson, CA, USA). Hysteresis loops were measured at room temperature under applied fields up to 2 T, while the magnetization measurements were performed in fields up to 5 T.

## 3. Results and Discussion

### 3.1. Formation and Decomposition of Glyoxylate Precursor and SiO_2_ Development

**Figure 1:**
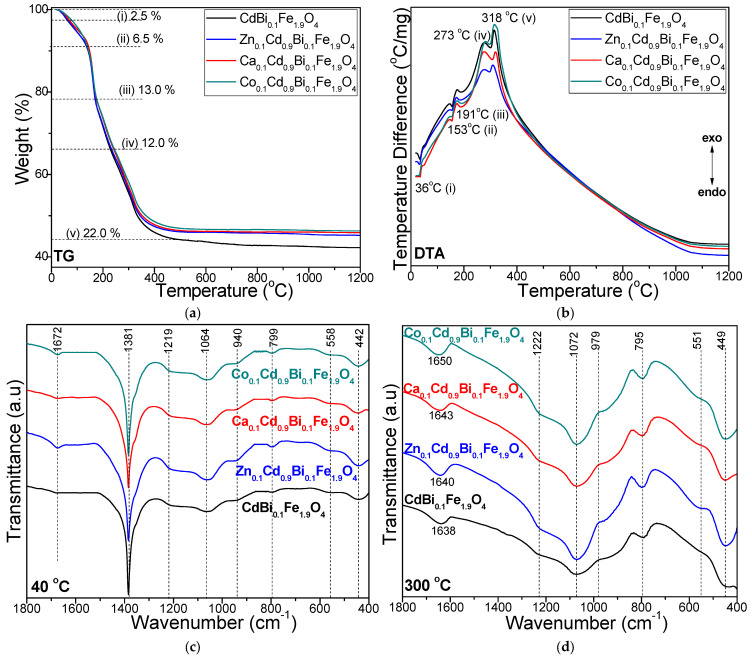
(**a**) TG and (**b**) DTA curves of CdBi_0.1_Fe_1.9_O_4_, Zn_0.1_Cd_0.9_Bi_0.1_Fe_1.9_O_4_, Ca_0.1_Cod_0.9_Bi_0.1_Fe_1.9_O_4_, Co_0.1_Cd_0.9_Bi_0.1_Fe_1.9_O_4_ gels heated at 40 °C; (**c**) FT-IR spectra of gels heated at 40 °C and (**d**) FT-IR spectra of gels calcinated at 300 °C.

Figure 1 presents the formation and decomposition of metal-glyoxylate precursors within the ferrite system, as well as the development of the SiO_2_ matrix, as evidenced by the TG-DTA profiles of the gels heated at 40 °C and the comparative FT-IR spectra of the gels heated at 40 and 200 °C.

The TG and DTA curves of all gels reveal five distinct thermal processes: (i) removal of residual water, including physically adsorbed moisture, manifested by an endothermic peak at approximatively 36 °C, corresponding to a mass loss of 2.5%; (ii) formation of Cd, Zn-, Ca-, Co- glyoxylates, evidenced by an endothermic effect near 153 °C with a mass loss of ~6.5%, accompanied by the release of H_2_O and NO (these redox reactions likely yield a homogenous mixture of homonuclear Fe^3+^, Bi^3+^ and Cd^2+^, Zn^2+^, Ca^2+^, Co^2+^ glyoxylates); (iii) formation of Fe- and Bi- glyoxylates occurring around 191 °C, marked by an endothermic effect and a mass loss of ~13%, due to the release of crystallization water from the nitrates and volatile species (H_2_O, NO_2_), driving redox reactions [13,15]; (iv) oxidative decomposition of Cd-, Zn-, Ca-, Co- glyoxylates into their corresponding metal oxides (CdO, ZnO, CaO, CoO) along with the concomitant formation of ferrites, appearing as an exothermic effect near 273 °C with the release of CO, CO_2_ and H_2_O, corresponding to a mass loss ~12%; and (v) oxidative decomposition of Fe- and Bi- glyoxylates into Fe_2_O_3_ and Bi_2_O_3_ occurring around 318 °C, producing an exothermic effect with a mass loss ~22% due to the elimination of CO, CO_2_ and H_2_O (Figure 1a,b) [13,14,15,28].

The two-stage progress (at 153 °C and 191 °C) of the redox reaction between metal nitrates and EG is attributed to the higher acidity of the [Fe(H_2_O)_6_]^3+^ and [Bi(H_2_O)_6_]^3+^ cations compared to [Cd(H_2_O)_6_]^2+^, [Zn (H_2_O)_6_]^2+^, [Ca(H_2_O)_6_]^2+^ and [Co(H_2_O)_6_]^2+^ cations [13,15]. Consequently, the redox reaction leading to the formation of the Fe carboxylates occurs at lower temperatures, producing a mixture of homonuclear Fe^3+^ and Co^2+^ carboxylates [13,15]. In contrast, the Fe^3+^ and Bi^3+^ glyoxylates form at higher temperatures [13,15]. All other synthesized gels display similar TG and DTA curves, with the position and intensity of the exothermic effects depending on the specific Fe(NO_3_)_3_/Bi(NO_3_)_3_/Co(NO_3_)_2_, Fe(NO_3_)_3_/Bi(NO_3_)_3_/Co(NO_3_)_2_/M^2+^(NO_3_) (M = Zn, Ca or Co) ratio. The highest total mass loss is observed for CdBi_0.1_Fe_1.9_O_4_@SiO_2_ (58.0%), while slightly lower mass losses are recorded for Zn_0.1_Cd_0.9_Bi_0.1_Fe_1.9_O_4_@SiO_2_ (54.7%), Ca_0.1_Cd_0.9_Bi_0.1_Fe_1.9_O_4_@SiO_2_ (54.3%), Co_0.1_Cd_0.9_Bi_0.1_Fe_1.9_O_4_@SiO_2_ (53.7%). During thermal treatment, the SiO_2_ matrix undergoes multiple transformations, which makes it difficult to make the distinction between processes associated with the formation and decomposition of glyoxylate precursors [13,14,15,28].

The FT-IR spectra indicate the formation of metal glyoxylate precursors from metal nitrates, as evidenced by the disappearance of the nitrate-related band at 1381 cm^−1^ upon calcination at 300 °C (Figure 1c,d) [13,14,15,28]. The SiO_2_ matrix already begins to develop at 40 °C and becomes more clearly defined after calcination at 300 °C, as confirmed by the presence of Si–O–Si stretching vibrations observed around 1064 cm^−1^ (40 °C) and 1072 cm^−1^ (300 °C), together with Si–O vibrational vibrations appearing as shoulders near 1219 cm^−1^ (40 °C) and 1222 cm^−1^ (300 °C) [13,14,15,16,23,24,25,26,27,28]. Deformation vibrations of the Si–OH groups, arising from the hydrolysis of –Si(OCH_2_CH_3_)_4_ (TEOS), are observed at 940 cm^−1^ (40 °C) and 972 cm^−1^ (300 °C), while Si–O stretching within SiO_4_ tetrahedra appear at 799 cm^−1^ (40 °C) and 795 cm^−1^ (300 °C) [13,14,15,16,23,24,25,26,27,28]. O–H vibrations from EG and adsorbed water appear at 1672 cm^−1^ at 40 °C; after calcination at 300 °C, these bands broaden and shift to 1638–1650 cm^−1^, increasing in intensity by Zn^2+^, Ca^2+^, or Co^2+^ doping. This behavior is attributed to –COO^−^ vibrations, indicating the formation of chelated complexes through coordination of glyoxylate ligands with the metal ions [13,14,15,16,23,24,25,26,27,28].

The FT-IR spectra exhibit absorption bands at 558 cm^−1^ (40 °C) and 551 cm^−1^ (300 °C), which are assigned to the stretching vibrations of tetrahedral Cd–O, Zn–O, Ca–O, and Co–O bonds, as well as to cyclic Si–O–Si structures. The bands observed at 442 and 449 cm^−1^ are associated with the octahedral Fe–O, Bi–O, and Si–O vibrational modes [13,14,15,16,23,24,25,26,27,28]. The shifts in the A- and B- site band positions with different dopants and calcination temperatures indicate lattice distortions arising from changes in M–O bond lengths [35,36]. These variations are in full agreement with the inverse relationship between bond length, reduced atomic mass, and stretching vibration frequency [37,38,39].

### 3.2. Formation of the Crystalline Structure of Ferrites

**Figure 2:**
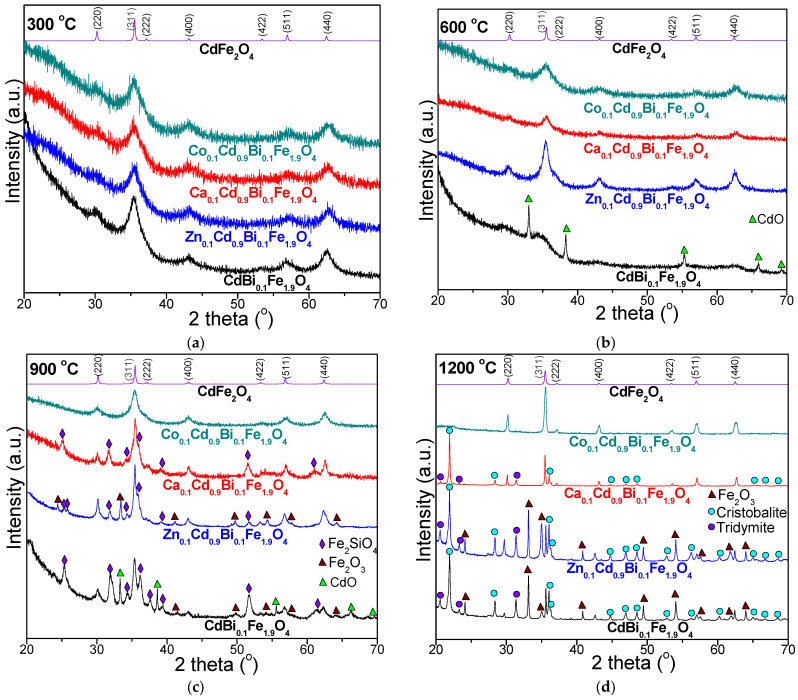
XRD patterns of CdBi_0.1_Fe_1.9_O_4_@SiO_2_, Zn_0.1_Cd_0.9_Bi_0.1_Fe_1.9_O_4_@SiO_2_, Ca_0.1_Cd_0.9_Bi_0.1_Fe_1.9_O_4_@SiO_2_, Co_0.1_Cd_0.9_Bi_0.1_Fe_1.9_O_4_@SiO_2_ NCs calcined at (**a**) 300 °C, (**b**) 600 °C, (**c**) 900 °C and (**d**) 1200 °C.

At low calcination temperatures, oxidic phases typically exhibit poor crystallinity or may remain partially amorphous; thus, the development of the desired surface characteristics and crystallinity strongly depends on optimizing the calcination conditions [13,14,16,19]. In addition, the pronounced chemical reactivity of the amorphous SiO_2_ matrix facilitates its participation in various chemical transformations during the calcination process. Figure 2 shows the XRD patterns of the NCs calcined at 300, 600, 900, and 1200 °C.

At 300 °C, the diffraction peaks corresponding to the (220), (311), (222), (400), (422), (511), and (440) planes confirm the formation of single, low-crystallized CdBi_0.1_Fe_1.9_O_4_ phase_,_ consistent with CdFe_2_O_4_ (JCPDS card no. 65-3115) and Bi_2_Fe_4_O_9_ (JCPDS card no. 74-1098), exhibiting a cubic spinel structure with space group *Fd3m* [26,27,28]. No secondary or impurity-phases are remarked at this temperature, indicating that the dopant ions are successfully incorporated into the spinel ferrite lattice without compromising its structural integrity.

At 600 °C, the XRD pattern of the undoped CdBi_0_._1_Fe_1_._9_O_4_@SiO_2_ NC is dominated by the CdO phase (JCPDS card no. 05-0640), while the characteristic diffraction peaks of the CdBi_0_._1_Fe_1_._9_O_4_ phase appear only weakly, indicating incomplete ferrite formation and poor crystallinity. In contrast, doping CdBi_0_._1_Fe_1_._9_O_4_ with Zn^2+^, Ca^2+^, and Co^2+^ ions promotes the formation of low crystallized single-phase ferrites. Among the doped samples, Zn_0_._1_Cd_0_._9_Bi_0_._1_Fe_1_._9_O_4_@SiO_2_ NC exhibits the most intense diffraction peaks, indicating the highest degree of crystallinity. These results suggest that incorporation of Zn^2+^, Ca^2+^, and Co^2+^ ions at 600 °C promotes the formation of single-phase ferrite structures and improves structural ordering compared with the undoped sample. The presence of secondary phases can be attributed to the higher cation mobility and lattice strain variations induced during calcination, which also slightly shift the 2θ positions, broadens the peaks, and increases crystallite sizes [37,40]. At 600 °C, doping CdBi_0.1_Fe_1.9_O_4_ with Zn^2+^, Ca^2+^, or Co^2+^ ions, restricts Cd^2+^ diffusion and enhances thermodynamic stability of Zn_0.1_Cd_0.9_Bi_0.1_Fe_1.9_O_4_, Ca_0.1_Cd_0.9_Bi_0.1_Fe_1.9_O_4_, Co_0.1_Cd_0.9_Bi_0.1_Fe_1.9_O_4_ phases embedded in SiO_2_ matrix. The homogeneity of the metal oxide particles may also contribute to an increased defect density and higher pore volume in the final composites [40,41].

At 900 °C, the XRD pattern of the undoped CdBi_0_._1_Fe_1_._9_O_4_@SiO_2_ NC reveals the coexistence of the crystalline ferrite phase with secondary crystalline phases of CdO, α-Fe_2_O_3_ (JCPDS card no. 33-0664), and Fe_2_SiO_4_ (JCPDS card no. 70-1861). For the Zn-doped CdBi_0_._1_Fe_1_._9_O_4_@SiO_2_ NC, CdO is no longer detected, while the diffraction pattern consists of the crystalline ferrite phase accompanied by α-Fe_2_O_3_ and Fe_2_SiO_4_. In the case of Ca-doped CdBi_0_._1_Fe_1_._9_O_4_@SiO_2_, α-Fe_2_O_3_ also disappears, resulting in a diffraction pattern containing the ferrite phase together with Fe_2_SiO_4_ as single secondary phase. The decomposition of carboxylate precursors generates a reducing environment that partially converts Fe^3+^ ions into Fe^2+^ ions, which subsequently interact with the SiO_2_ matrix, resulting in the formation of Fe_2_SiO_4_ at 900 °C in all NCs [27,28,39]. The formation of α-Fe_2_O_3_ instead of pure crystalline ferrite may result from the incomplete embedding of the ferrite within the SiO_2_ matrix and from suboptimal calcinations conditions [19,20]. The presence of α-Fe_2_O_3_ indicates the decomposition of Fe(NO_3_)_3_ into α-Fe_2_O_3_, which promotes spinel ferrite formation, while the excess of metal oxides in insoluble secondary phases may enhance densification by increasing pore volume and generating demagnetizing fields [37,40]. The formation of secondary phases is further influenced by the cation mobility and the lattice strain induced during the calcination process. These effects also display as peak broadening, increased crystallite size, and slight shifts in 2θ positions [14,27,28].

Following calcination at 600 and 900 °C, the ferrite phases remain poorly crystalline, while higher calcination temperatures promote the formation of larger crystallites and accelerate the crystallite growth due to improved nucleation and growth kinetics [27,28]. The presence of sharp diffraction peaks at elevated temperature confirms the development of well-crystallized spinel ferrite structures. At 1200 °C, both undoped CdBi_0_._1_Fe_1_._9_O_4_@SiO_2_ and Zn_0_._1_Cd_0_._9_Bi_0_._1_Fe_1_._9_O_4_@SiO_2_ display four crystalline phases: CdBi_0_._1_Fe_1_._9_O_4_, α-Fe_2_O_3_, cristobalite (JCPDS card no. 74-9378), and tridymite (JCPDS card no. 42-1401), indicating partial crystallization of the SiO_2_ matrix in the absence of ferrite. Ca^2+^ doping of CdBi_0_._1_Fe_1_._9_O_4_@SiO_2_ leads to the disappearance of the α-Fe_2_O_3_ phase, resulting in the ferrite phase coexisting only with the crystalline SiO_2_ phases (cristobalite and tridymite). In contrast, Co^2+^ doping results in the formation of a single-phase ferrite, Co_0_._1_Cd_0_._9_Bi_0_._1_Fe_1_._9_O_4_, corresponding to CoFe_2_O_4_ (JCPDS card no. 22-1086), CdFe_2_O_4_ (JCPDS card no. 65-3115), and Bi_2_Fe_4_O_9_ (JCPDS card no. 74-1098). This behavior is attributed to the atomic level cation mixing, which promotes the formation of a dense, fine-grained, and homogeneous ferrite structure [40,41,42,43].

The diffraction peaks of the ferrite phases become more intense at 1200 ◦C, indicating high crystallinity, uniform nucleation, and efficient crystal growth, with minimal influence of from surface coatings [14,27,28]. Variations in peak intensity and signal-to-noise ratio corresponds to the variations in crystallite size and degree of crystallinity [32].

Table 1 summarizes the structural parameters of the NCs calcined at 300, 600, 900 and 1200 °C, including average crystallite size (D_CS_), lattice parameter (a), unit cell volume (V), physical (d_p_) and X-ray (d_XRD_) densities, hopping length at the A (d_A_) and B (d_B_) sites, and porosity (P), as determined by XRD [44,45].

Across all calcination temperatures, the undoped CdBi_0_._1_Fe_1_._9_O_4_ exhibits the smallest D_CS_, whereas doping with Zn^2+^, Ca^2+^, and Co^2+^ ions lead to a noticeable increase in D_CS_ (Table 1). The calcination temperature-driven growth of D_CS_ can be explained by (i) elevated thermal energy, which enhances atomic diffusion, promotes particle coalescence, and favors the formation of larger crystallites; (ii) agglomeration of incompletely crystallized particles, which reduces the effective nucleation density and supports the formation of single, well-defined crystals; and (iii) variations in cation valence and ionic radii, which induce lattice strain, generate additional nucleation sites, and facilitate crystallite formation [14,27,28]. Additionally, peak broadening, lattice strain and the constraining effect of the SiO_2_ matrix can further modulate the crystallite growth. D_CS_ is a key determinant of the magnetic behavior of the NCs, particularly when the particle size nears the crystallite size [14,23,24]. These results highlight that the synthesis strategy and calcination conditions critically influence the structural and magnetic characteristics of the NCs. The Reference Intensity Ratio (RIR) method was used to determine the quantitative phase analysis (Table 1) [45,46].

The lattice parameter (a) exhibits the highest values in undoped CdBi_0_._1_Fe_1_._9_O_4_ across all calcination temperatures. Doping with Zn^2+^, Ca^2+^, or Co^2+^ ions leads to a reduction in the lattice parameter, ascribed to the smaller ionic radii of the dopants, namely Zn^2+^ (0.74 Å) [3,9,46,47], Ca^2+^ (0.95 Å) [39], Co^2+^ (0.75 Å) [19,21,25] compared to Cd^2+^ (0.97 Å) [4,5,10,12]. The lattice parameter increases with calcination temperature from 8.344 to 8.498 Å, reflecting the combined effect of ionic radii, lattice distortions, and structural defects [14,16,23,27]. Differences between experimental and theoretical lattice parameters arise from the idealized assumption that ions behave as rigid spheres within a perfectly ordered lattice. In the CdBi_0_._1_Fe_1_._9_O_4_ structure, the Cd^2+^ ions and dopant (Zn^2+^, Ca^2+^ and Co^2+^) ions predominantly occupy the A- sites, whereas Fe^3+^ and Bi^3+^ ions are distributed across both A- and B- sites, depending on the degree of cation disorder and specific synthesis conditions [10,17,18]. Lattice shrinkage is primarily governed by the cation charge distribution, surface tension, and dipole interactions within the nanocrystallites, which also limit grain growth. The internal stresses associated with variations in the lattice parameter can hinder grain development further during calcination [16,23,27].

The cell volume (V) gradually increases with calcination temperature, ranging from 581 to 614 Å^3^. Doping CdBi_0.1_Fe_1.9_O_4_@SiO_2_ with Zn^2+^, Ca^2+^, Co^2+^ ions causes a slight reduction in cell volume, likely due to the combined effects of higher molecular mass, the formation of oxygen vacancies, and decreased crystallization temperature. These factors jointly offset the expected volumetric contraction associated with the densification [14,27,28].

The hopping lengths for the tetrahedral A (d_A_) and octahedral B (d_B_) sites are closely correlated to the lattice parameter. Lattice expansion is primarily driven by the greater ionic radii of Cd^2+^, Ca^2+^, and Bi^3+^ ions compared to those of Zn^2+^, Co^2+^, and Fe^3+^ ions. Moreover, the Cd^2+^ ions and the dopant ions (Zn^2+^, Ca^2+^, Co^2+^) show a limited preference for the A sites, whereas Fe^3+^ and Bi^3+^ ions are unequally distributed between the A- and B- sites, depending on the composition of undoped and doped CdBi_0_._1_Fe_1_._9_O_4_@SiO_2_ [14,27,28].

Higher d_A_ and d_B_ indicate greater cation separation, which increases the energy barrier for charge carrier transfer and influences the magnetic characteristics. Since the crystal field splitting at (A) sites is smaller than at the B sites, variations in cation distribution, particularly of Zn^2+^, Ca^2+^, Co^2+^, Cd^2+^, and Fe^3+^ ions, and changes in the degree of inversion can modify the 3d orbitals energy levels, resulting in band gap modifications [14,27,28,45]. Furthermore, replacing magnetic Cd^2+^ ions with Zn^2+^, Ca^2+^, or Co^2+^ ions adjust the cation distribution within the ferrite lattice, thereby affecting the A-B superexchange interactions, and enabling tunable magnetic behavior [45].

The d_XRD_ reflects the dislocation density per unit volume in a crystalline material [11,13] and is primarily influenced by the lattice parameter and molecular mass [15]. In this study, the d_XRD_ increases with Zn^2+^, Ca^2+^, Co^2+^ ions doping in CdBi_0.1_Fe_1.9_O_4_@SiO_2_ NCs, whereas it gradual decrease with the increasing calcination temperature (from 6.855 to 6.513 g/cm^3^), which could be attributed to the pore formation during the thermal treatment [15]. Variations in d_XRD_ associated with changes in the lattice parameter are attributed to modified cation distribution between the A- and B- sites. Replacing Cd^2+^ ions with Zn^2+^, Ca^2+^, or Co^2+^ ions reduces grains compactness and promotes grain growth, likely due to the pore development through calcination and subsequent sample processing. Additionally, disparities in ionic radii introduce lattice strain and structural disorder, thereby perturbing the spinel framework of the ferrite [11,13].

The porosity (P) of undoped CdBi_0.1_Fe_1.9_O_4_@SiO_2_ (22.1–16.7%) was higher than that of Zn_0.1_Cd_0.9_Bi_0.1_Fe_1.9_O_4_@SiO_2_ (21.7–16%), Ca_0.1_Cd_0.9_Bi_0.1_Fe_1.9_O_4_@SiO_2_ (20.2–14.5%), Co_0.1_Cd_0.9_Bi_0.1_Fe_1.9_O_4_@SiO_2_ (21.2–15.6%) NCs. This trend is attributed to enhanced vacancy generation and pore formation during synthesis, whereas higher calcination temperatures facilitate densification, resulting in a progressive reduction in porosity [48,49]. Low porosity, as observed for Co_0.1_Cd_0.9_Bi_0.1_Fe_1.9_O_4_@SiO_2_ calcined at 1200 °C, is essential for achieving superior magnetic performance, whereas higher porosity levels can negatively affect magnetic behavior [14,27,28,48,49]. Additionally, doping with Zn^2+^, Ca^2+^, Co^2+^ ions influences porosity by promoting the formation of oxygen vacancies, which slightly expand the lattice while maintaining the structural symmetry of the ferrite framework [14,27,28,48,49].

### 3.3. AFM Analysis

XRD analysis indicates that the calcination temperatures above 600 °C promotes the development of well-defined ferrite crystallites, while the samples calcined at 300 °C remain amorphous. High calcination temperatures also enhance the tendency of adjacent particles to coalesce. Such behavior has been reported for fine clay particles [50], where physical attraction without chemical bonds governs aggregation; dispersion in an aqueous medium ensures proper particle separation and individualization [51]. Thus, the NCs calcined at 600, 900 and 1200 °C were dispersed into the ultra-pure water by magnetic agitation at 3000 rpm for 15 min. Afterward, glass slides were vertically immersed into the dispersions, allowing the Brownian motion of nanoparticles to promote adsorption onto the slide surface. The literature indicates that an adsorption time of 10–30 s is sufficient to ensure a uniform a thin film of nanoparticles transferred onto the solid substrate [51]. Following this guideline, the ferrite nanoparticles were adsorbed for 15 s, followed by the gentle extraction of the glass slide followed by natural drying. This procedure ensures a proper individualization of the adsorbed nanoparticles and results in thin films with topographical features that depend on the sample’s composition.

**Figure 3:**
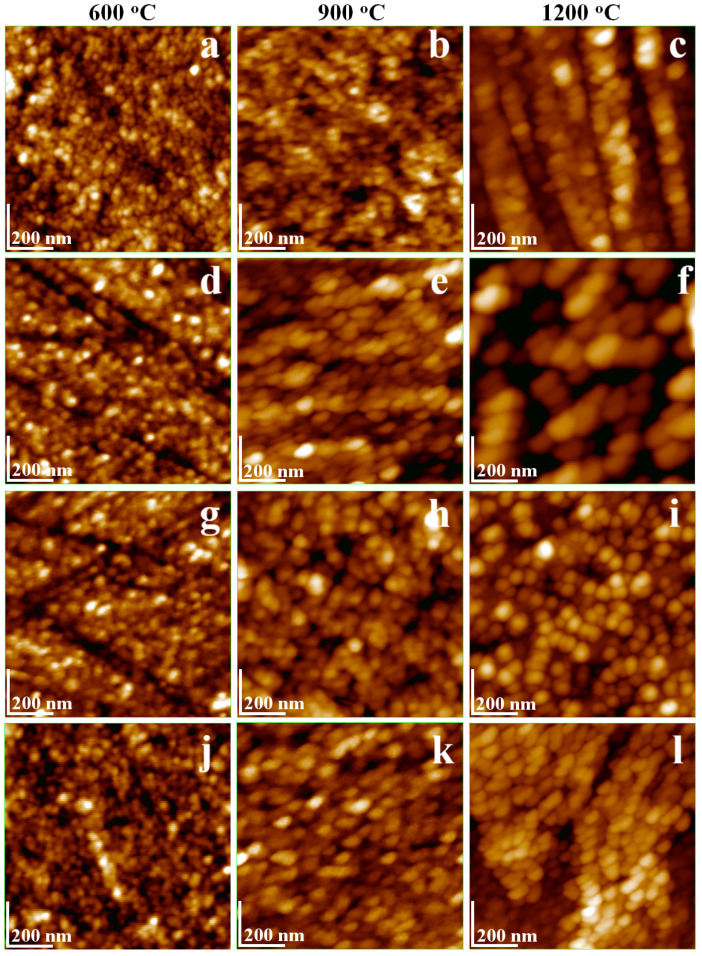
AFM topographic images of the powders’ thin films transferred onto solid substrate: CdBi_0.1_Fe_1.9_O_4_@SiO_2_ NC calcined at (**a**) 600 °C, (**b**) 900 °C, (**c**) 1200 °C; Zn_0.1_Cd_0.9_Bi_0.1_Fe_1.9_O_4_@SiO_2_ NC calcined at (**d**) 600 °C, (**e**) 900 °C, (**f**) 1200 °C; Ca_0.1_Cd_0.9_Bi_0.1_Fe_1.9_O_4_@SiO_2_ NC calcined at (**g**) 600 °C, (**h**) 900 °C, (**i**) 1200 °C; Co_0.1_Cd_0.9_Bi_0.1_Fe_1.9_O_4_@SiO_2_ NC calcined at (**j**) 600 °C, (**k**) 900 °C, (**l**) 1200 °C.

The CdBi_0.1_Fe_1.9_O_4_@SiO_2_ consists of very small, rounded nanoparticles after calcination at 600 °C, with a diameter of 18 nm (Figure 3a). This diameter is slightly larger than the corresponding crystallite size of 14.9 nm, which can be attributed to the amorphous SiO_2_ shell embedding the ferrite crystallite as the composite core. Ferrite core increases with the calcination temperature developing the rectangle shape characteristics of its crystal structure. Thus, the particles calcined at 900 °C exhibit partial alteration of the rounded shape due to the ferrite core growth alongside the amorphous SiO_2_ coating, leading to a slight increase in diameter to about 35 nm (Figure 3b). This trend is further accentuated after calcination at 1200 °C (Figure 3c) where the nanoparticles become slightly elongated due to the extensive development of the ferrite crystallite core, resulting in diameters up to 52 nm. These observations are consistent with literature reports on CdFe_2_O_4_ [52], where nanoparticle elongation is evident in SEM images [53]. Moreover, the perovskite structure of BiFeO_3_ has a square-to-elongated crystal lattice, in agreement with the AFM observations [54], with particle sizes increasing with the calcination temperature [55].

Previous studies have shown that doping BiFeO_3_ with Cd^2+^ and Mg^2+^ ions significantly improves its microstructural and magnetic properties by stabilizing the spinel phase, thereby enhancing both dielectric and magnetic performance [56]. The AFM images indicate a synergistic effect of Cd and Bi substitution on the growth and development of the ferrite core. Nayak et.al emphasizes that structural changes within BiFeO_3_ play a key role in tailoring its magnetic behavior [57]. Building on this, we propose that additional tuning of the ferrite characteristics can be achieved through the strategic incorporation of minor dopants. This hypothesis is supported by the observed improvements in the crystal lattice, as observed in XRD patterns presented in Figure 2.

Zn^2+^ doping of CdBi_0.1_Fe_1.9_O_4_ exhibits a pronounced effect, promoting well-developed particles even at a relatively low calcination temperature (600 °C, Figure 3d). The nanoparticles display a diameter of approximately 22 nm and a predominantly rounded shape. The increase in calcination temperature facilitates optimal growth of the ferrite core, which alters the nanoparticle morphology: the particles become slightly elongated after calcination at 900 °C and develop a blockier, boulder-like shape with softened square corners after calcination at 1200 °C, partially moderated by the amorphous SiO_2_ shell (Figure 3e,f). A similar evolution in morphology is observed on the NCs doped with Ca^2+^ (Figure 3g–i).

Co^2+^ doping of CdBi_0.1_Fe_1.9_O_4_ exhibit hybrid behavior, revealing small, rounded nanoparticles after calcination at 600 °C (Figure 3j) with a diameter of approximatively 19 nm, closely resembling the undoped CdBi_0.1_Fe_1.9_O_4_. The doping effect on the nanoparticle structure becomes apparent after calcination at 900 °C (Figure 3k), inducing a significant increase of the particle size to about 38 nm and pronounced elongation due to the enhanced growth of the ferrite core. Further increasing the calcination temperature increases to 1200 °C leads to only a minor increase in particle diameter to 54 nm, but evidence a strong consolidation of the ferrite core, ensuring optimal stability and well-defined individualization of the NC structure.

Considering the approximate rounded shape of the nanoparticles and ferrite crystallite cores, the thickness of the amorphous SiO_2_ shell is estimated to about 2 nm. This value is consistent with the expectations of the synthesis procedure. It should be noted, however, that AFM cannot reliably differentiate between the ferrite core and the amorphous SiO_2_ shell, whereas XRD analysis allows for this differentiation. This represents a limitation of the present study.

**Figure 4:**
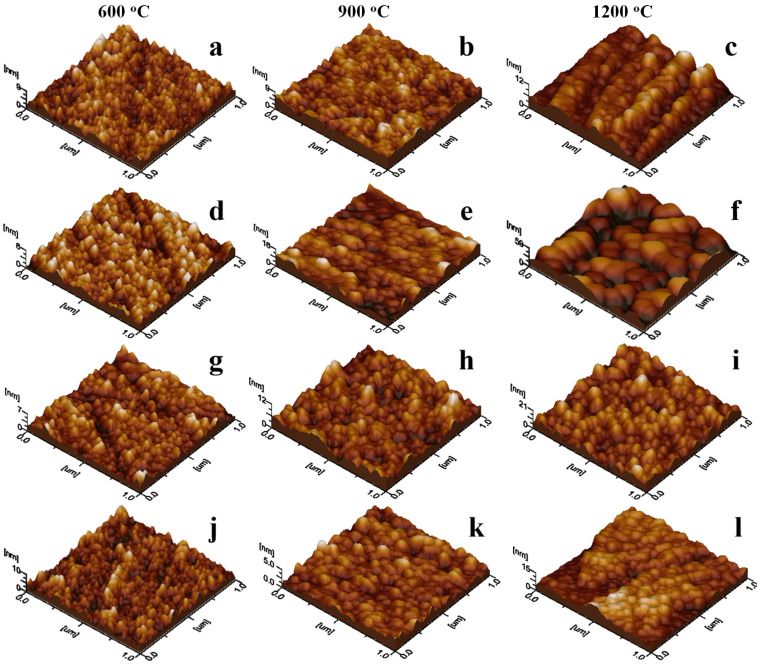
AFM tridimensional profiles of the powders’ thin films transferred onto solid substrate: CdBi_0.1_Fe_1.9_O_4_@SiO_2_ NC calcined at (**a**) 600 °C, (**b**) 900 °C, (**c**) 1200 °C; Zn_0.1_Cd_0.9_Bi_0.1_Fe_1.9_O_4_@SiO_2_ NC calcined at (**d**) 600 °C, (**e**) 900 °C, (**f**) 1200 °C; Ca_0.1_Cd_0.9_Bi_0.1_Fe_1.9_O_4_@SiO_2_ NC calcined at (**g**) 600 °C, (**h**) 900 °C, (**i**) 1200 °C; Co_0.1_Cd_0.9_Bi_0.1_Fe_1.9_O_4_@SiO_2_ NC calcined at (**j**) 600 °C, (**k**) 900 °C, (**l**) 1200 °C.

Amorphous SiO_2_ shell provides an optimal insulation of the ferrite nuclei during the ferrite crystallization, preventing the coalescence between adjacent crystallites. This facilitates the optimal encapsulation of the ferrite core into well individualized composite nanoparticles [58,59,60]. The optimal encapsulation promotes uniform dispersion of the nanoparticles into the aqueous environment which favors the uniform adsorption onto the solid substrate (e.g., glass slide) (Figure 4). The roughness of the surface strongly depends on the nanoparticle’s diameter and the uniformity of adsorbed layer. For example, the samples calcined at 600 °C exhibit low roughness and minimal local height variations ensuring a thin and compact film (Figure 4a,d,g,j). The continuous growth of the ferrite core partly alters the nanoparticle shapes (Figure 4b,e,h,k) leading to the relative increase of the surface roughness (Table 2). The slightly roughness enhancement facilitates the nanoparticle functionalization with biomedical agents (e.g., anticancer drug for targeted delivery onto the tumor sites) according to the literature data [61,62]. Moreover, the higher calcination temperature promotes optimal crystallization of the ferrite core and further increases surface roughness due to the adsorption of larger and more irregularly shaped nanoparticles. Despite this, the resulting thin films remain relatively uniform owing to the well-individualized nanoparticles, which prevent coalescence. The literature highlights the versatility and potential of such ferrites due to their structural stability in composite materials, while also noting associated environmental concerns [63]. In our study, the well-dispersed nanoparticles observed in the adsorbed thin films enable efficient recovery from environmental media via magnetic separation, allowing applications such as oil spill remediation [64] or degradation of dye pollutants [65].

### 3.4. Magnetic Behavior

**Figure 5:**
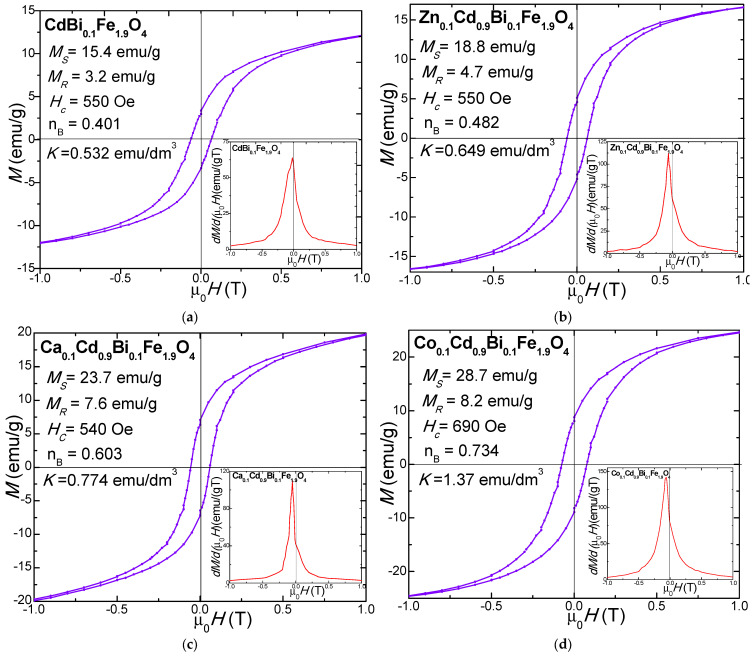
Magnetic hysteresis loops and magnetization derivative of (**a**) CdBi_0.1_Fe_1.9_O_4_@SiO_2_, (**b**) Zn_0.1_Cd_0.9_Bi_0.1_Fe_1.9_O_4_@SiO_2_, (**c**) Ca_0.1_Cd_0.9_Bi_0.1_Fe_1.9_O_4_@SiO_2_, and (**d**) Co_0.1_Cd_0.9_Bi_0.1_Fe_1.9_O_4_@SiO_2_ NCs calcined at 1200 °C.

The magnetic hysteresis loops *M(µ_0_H)* and their first derivatives *(dM/d(μ_0_H)* of NCs calcined at 1200 °C exhibit typical soft ferrimagnetic behavior (Figure 5). At the nanoscale, the high surface-to-volume ratio positions many atoms at or near the particle surface, where broken bonds, defects, spin disorder, and spin canting generate surface spin effects that reduce magnetization and broaden the *H_c_* distribution [66]. As surface contributions increase, surface-dominated interactions outweigh core effects, causing deviations from bulk behavior. Thus, the magnetic characteristics are strongly governed by particle size, shape, and morphology, which depend on synthesis and thermal treatment conditions [27,28,66,67].

All samples exhibit characteristic *S*-shaped hysteresis loops (Figure 5), which indicated that relatively high magnetocrystalline anisotropy (*K*) requires stronger fields for saturation. The moderate *H_c_* observed is attributed to partial particle coalescence, which enhances interparticle magnetic coupling and overall magnetization. However, diminished magnetic performance may result from a magnetically inactive surface layer associated with defects, altered cation distribution, incomplete saturation, and lattice defects [14,16,27].

The derivative curves *dM/d(μ_0_H)* vs. *μ0H* reveal magnetization reversal dynamics. For the NCs calcined at 1200 °C, a dominant peak near *H_c_* indicates a single magnetic phase [14,16,23], while a broad peak reflects a distribution of particle sizes and *H_c_*. The minimal peak shift from the origin indicates a narrow *H_c_* distribution due to strong interparticle coupling [68,69]. Sharp peaks are associated with well-crystallized, magnetically homogeneous samples, whereas broader features indicate increased size dispersion and internal defects [14,27]. These results confirm the formation of uniform, well-crystallized nanocrystals.

Doping CdBi_0.1_Fe_1.9_O_4_@SiO_2_ with non-magnetic Zn^2+^, Ca^2+^, and Co^2+^ ions modifies the magnetocrystalline anisotropy and particle size, leading to enhanced *M_S_*, remnant magnetization (*M_R_*), magnetic moments per unit cell (n_B_), *H_C_* and *K* compared to undoped CdBi_0.1_Fe_1.9_O_4_ [14,27,28]. This enhancement is attributed to the dopant ions which promote soft ferrimagnetic behavior [14,16,23,27,67].

*M_s_* of ferrites is primarily governed by the superexchange interactions between the A- and B- sites. An increase in D_c_ enhances *M_s_* by reducing surface-related defects and improving magnetic ordering [67]. In this study, the highest *M_s_* value (28.7 emu/g) was observed for Co_0.1_Cd_0.9_Bi_0.1_Fe_1.9_O_4_@SiO_2_ NC, whereas the undoped CdBi_0.1_Fe_1.9_O_4_@SiO_2_ NC exhibited lower *M_s_* values (15.4 emu/g). The surface phenomena such as broken chemical bonds, deviations from the ideal cation distribution, lattice defects, and randomly oriented magnetic moments, contribute to reducing the overall magnetic performance. Additionally, the structural disorder at the particle surface can disrupt the alignment of magnetic moments, a phenomenon typically associated with single-domain configurations [14,16,23,27,67]. Partial substitution of Cd^2+^ with Zn^2+^, Ca^2+^, Co^2+^ ions may also reduce *M_s_* due to the spin canting arising from the triangular spin configuration within the sublattice, as described by the Yaffet–Kittel [14,16,23,27]. Overall, the enhanced characteristics of Co_0.1_Cd_0.9_Bi_0.1_Fe_1.9_O_4_@SiO_2_ NC, including its relatively high *Ms* and favorable combination of structural and morphological characteristics, make it a promising candidate for technological applications in communication devices [22,26,32].

The remanent magnetization (*M_R_*) of undoped CdBi_0.1_Fe_1.9_O_4_@SiO_2_ is 3.2 emu/g. The doping with Zn^2+^ ions increases *M_R_* to 4.7 emu/g, while Co^2+^ ions doping results in an even greater increase, reaching 8.2 emu/g, demonstrating the significant effect of the dopant type on the *M_R_* of ferrites.

The *K* value was calculated under the assumption that the spinel ferrite nanoparticles are approximatively spherical. The *K* is governed by crystal symmetry, intrinsic magneto crystalline anisotropy, as well as particle size and shape [14,16,23,27]. Among the studied NCs calcined at 1200 °C, Co_0.1_Cd_0.9_Bi_0.1_Fe_1.9_O_4_@SiO_2_ NC exhibits the highest K value of 1.34 erg/dm^3^. The increase in *K* for the CdBi_0.1_Fe_1.9_O_4_@SiO_2_ NC with the addition of dopant ions, reaching the highest value for Co_0.1_Cd_0.9_Bi_0.1_Fe_1.9_O_4_@SiO_2_ NC, follows a trend similar to *M_S_*, although no clear linear or colinear correlation between *M_S_* and *K* is observed. Generally, K values increase with doping, which can be attributed to several factors: (i) partial pinning of surface spins in magnetically disordered surface layers, requiring stronger magnetic fields to achieve saturation, and (ii) the presence of randomly oriented grains and lattice vacancies, which further contribute to magnetic disorder. Additionally, crystalline *K* is affected by the lattice strain arising from the partial substitution of Cd^2+^ by Zn^2+^, Ca^2+^, or Co^2+^ ions, whichalters the local structural environment and contributes to *K* enhancement [14,16,23,27].

The lowest n_B_ value is observed in undoped CdBi_0.1_Fe_1.9_O_4_ (0.401) increasing upon doping with Zn^2+^ (0.482), Ca^2+^ (0.603) or Co^2+^ (0.734) doping, which indicates an enhancement of non-collinear spin structures as described by the Yafet–Kittel model. The low M_s_ in undoped CdBi_0.1_Fe_1.9_O_4_ is attributed to spin canting in the B- sublattice, Jahn-Teller cations, surface magnetic dead layers, cation distribution deviations, incomplete saturation, random moment orientation, and lattice defects. Magnetization in larger particles is dominated by domain wall motion, while smaller particles form single domains aligned with the applied field [69].

The modest increase in *H_c_* values for the doped NCs can be attributed to the contributions from magnetocrystalline anisotropy, microstrain effects, particle size distribution, and reduction in magnetic domain dimensions [31]. The undoped CdBi_0.1_Fe_1.9_O_4_@SiO_2_ NC exhibits an *H_c_* value of 530 Oe, whereas Co_0.1_Cd_0.9_Bi_0.1_Fe_1.9_O_4_@SiO_2_ NC exhibit the highest *H_C_* of 690 Oe. This enhancement is likely related to the variations in D_c_, *K* and the formation of particle agglomerates that increase the particle size beyond the single-domain threshold, resulting in multi-domain structures and reduced domain wall pinning at grain boundaries [14,16,23,27,67]. Conversely, lower *H_c_* values suggest surface spin distortion arising from variations in the magnetocrystalline *K*. In addition, an increased density of surface lattice defects, such as the atom displacement in the surface layers, could develop local energy barriers that further contribute to increased *H_C_* [14,16,23,27,67].

Embedding of magnetic ferrites within the SiO_2_ matrix stabilizes and organizes the magnetic domains, leading to well-defined magnetic nano-crystallites within the NC. This structural reinforcement improves the mechanical integrity and processability, enabling potential applications in magnetic coatings, thin films, and other nanostructured devices [2,3,6,32].

## 4. Conclusions

The influence of Zn^2+^, Ca^2+^, Co^2+^ ions doping on the structural, morphological, surface, and magnetic characteristics of CdBi_0.1_Fe_1.9_O_4_ was systematically investigated. At 300 °C, the ferrites exhibited poorly crystallinity, whereas Co_0.1_Cd_0.9_Bi_0.1_Fe_1.9_O_4_@SiO_2_ calcined at 1200 °C displayed a well-defined single-phase ferrite structure. Doping not only enhanced ferrite crystallinity but also promoted the development of crystalline SiO_2_ phases (silicate, cristobalite, and tridymite). Although all NCs maintained a cubic spinel structure, XRD analysis revealed that the dopant incorporation induced variations in the structural parameters. AFM imaging confirmed that the ferrite crystallites observed by XRD are encapsulated within amorphous SiO_2_, forming well-individualized nanoparticles. At lower calcination temperatures, the nanoparticles are predominantly rounded, whereas higher calcination temperatures enhance ferrite core growth, leading to distorted particle shapes and increased sizes. The surface roughness of the adsorbed thin films strongly correlates with nanoparticle size and morphology: small, rounded particles yield low roughness, while larger, irregular nanoparticles significantly increase surface roughness, making them suitable for targeted functionalization. The *M_S_* (15.4–28.7 emu/g), *M_R_* (3.2–8.2 emu/g), *H_c_* (530–690 Oe) and K (0.532–1.24 erg/dm^3^) diminished with the incorporation of dopant ions, approaching soft ferrimagnetic-like behavior. The analysis using Néel’s collinear and Yafet–Kittel spin-canting models, considering the surface spin effects, indicates that doping and thermal treatment are critical factors in governing the magnetic behavior and particle size distribution of the nanocomposites.

## Figures and Tables

**Table 1:** Structural parameters and quantitative analysis of CdBi_0.1_Fe_1.9_O_4_@SiO_2_, Zn_0.1_Cd_0.9_Bi_0.1_Fe_1.9_O_4_@SiO_2_, Ca_0.1_Cd_0.9_Bi_0.1_Fe_1.9_O_4_@SiO_2_, Co_0.1_Cd_0.9_Bi_0.1_Fe_1.9_O_4_@SiO_2_ NCs estimated by XRD.

Sample	Temp [°C]	D_CS_ [nm]	a (Å)	V (Å^3^)	d_A_ (Å)	d_B_ (Å)	d_p_ (g/cm^3^)	d_XRD_ (g/cm^3^)	P (%)	Quantitative Analysis
CdBi_0.1_Fe_1.9_O_4_@SiO_2_	300	7.3	8.379	588	3.628	2.962	5.342	6.855	22.1	100% CdBi_0.1_Fe_1.9_O_4_
	600	14.9	8.415	596	3.644	2.975	5.379	6.763	20.5	74% CdO/26% CdBi_0.1_Fe_1.9_O_4_
	900	31.6	8.457	605	3.662	2.990	5.411	6.662	18.8	34%CdBi_0.1_Fe_1.9_O_4_/23% Fe_2_SiO_4_/6%Fe_2_O_3_/37%CdO
	1200	48.2	8.498	614	3.680	3.004	5.467	6.564	16.7	32% CdBi_0.1_Fe_1.9_O_4_/35% Fe_2_O_3_/33% SiO_2_
Zn_0.1_Cd_0.9_Bi_0.1_Fe_1.9_O_4_@SiO_2_	300	8.3	8.344	581	3.613	2.950	5.349	6.830	21.7	100% Zn_0.1_Cd_0.9_Bi_0.1_Fe_1.9_O_4_
	600	16.5	8.376	588	3.627	2.961	5.393	6.749	20.0	100% Zn_0.1_Cd_0.9_Bi_0.1_Fe_1.9_O_4_
	900	34.8	8.423	598	3.648	2.978	5.431	6.636	18.2	73% Zn_0.1_Cd_0.9_Bi_0.1_Fe_1.9_O_4_/ 22% Fe_2_SiO_4_/5%Fe_2_O_3_
	1200	52.4	8.468	607	3.667	2.994	5.489	6.538	16.0	34% Zn_0.1_Cd_0.9_Bi_0.1_Fe_1.9_O_4/_35% Fe_2_O_3_/31% SiO_2_
Ca_0.1_Cd_0.9_Bi_0.1_Fe_1.9_O_4_@SiO_2_	300	9.6	8.366	586	3.623	2.958	5.359	6.714	20.2	100% Ca_0.1_Cd_0.9_Bi_0.1_Fe_1.9_O_4_
	600	15.9	8.406	594	3.640	2.972	5.408	6.624	18.4	100% Ca_0.1_Cd_0.9_Bi_0.1_Fe_1.9_O_4_
	900	36.2	8.446	603	3.657	2.986	5.449	6.525	16.5	72% Ca_0.1_Cd_0.9_Bi_0.1_Fe_1.9_O_4_/18% Fe_2_SiO_4_
	1200	56.5	8.488	612	3.675	3.001	5.497	6.429	14.5	71% Ca_0.1_Cd_0.9_Bi_0.1_Fe_1.9_O_4_/29% SiO_2_
Co_0.1_Cd_0.9_Bi_0.1_Fe_1.9_O_4_@SiO_2_	300	7.9	8.353	583	3.617	2.953	5.352	6.792	21.2	100% Co_0.1_Cd_0.9_Bi_0.1_Fe_1.9_O_4_
	600	15.7	8.389	590	3.633	2.966	5.398	6.711	19.6	100% Co_0.1_Cd_0.9_Bi_0.1_Fe_1.9_O_4_
	900	33.0	8.435	600	3.652	2.982	5.440	6.599	17.6	100% Co_0.1_Cd_0.9_Bi_0.1_Fe_1.9_O_4_
	1200	49.8	8.473	608	3.669	2.996	5.495	6.513	15.6	100% Co_0.1_Cd_0.9_Bi_0.1_Fe_1.9_O_4_

**Table 2:** Topographic parameters and particle diameter measured with AFM.

Sample	Temp, °C	Height, nm	Ra, nm	D, nm
CdBi_0.1_Fe_1.9_O_4_@SiO_2_	600	9	0.89	18
	900	9	1.14	35
	1200	12	1.87	52
Zn_0.1_Cd_0.9_Bi_0.1_Fe_1.9_O_4_@SiO_2_	600	8	0.86	22
	900	16	2.25	43
	1200	59	9.06	72
Ca_0.1_Cd_0.9_Bi_0.1_Fe_1.9_O_4_@SiO_2_	600	7	0.90	20
	900	12	1.54	41
	1200	21	3.40	64
Co_0.1_Cd_0.9_Bi_0.1_Fe_1.9_O_4_@SiO_2_	600	10	1.10	19
	900	5	0.63	38
	1200	15	2.44	54

## Data Availability

Data are available on request from the corresponding author.
